# Deep analysis of acquired resistance to FGFR1 inhibitor identifies MET and AKT activation and an expansion of *AKT1* mutant cells

**DOI:** 10.18632/oncotarget.25862

**Published:** 2018-07-31

**Authors:** Pol Gimenez-Xavier, Eva Pros, Ana Aza, Sebastian Moran, Raul Tonda, Anna Esteve-Codina, Marc Dabad, Montse Sanchez-Cespedes

**Affiliations:** ^1^ Genes and Cancer Group, Cancer Epigenetics and Biology Program (PEBC), Bellvitge Biomedical Research Institute-IDIBELL, Hospitalet de Llobregat, Barcelona, Spain; ^2^ Cancer Epigenetics Group, Cancer Epigenetics and Biology Program (PEBC), Bellvitge Biomedical Research Institute-IDIBELL, Hospitalet de Llobregat, Barcelona, Spain; ^3^ CNAG-CRG, Centre for Genomic Regulation (CRG) and Institute of Science and Technology (BIST), Barcelona, Spain; ^4^ Universitat Pompeu Fabra (UPF), Barcelona, Spain

**Keywords:** lung cancer, FGFR1, acquired resistance, tyrosine kinase inhibitor, cell lines

## Abstract

The development of acquired resistance (AR) to tyrosine kinase inhibitors (TKIs) of FGFR1 activation is currently not well understood. To gain a deeper insight into this matter in lung cancer, we used the FGFR1-amplified DMS114 cell line and generated multiple clones with AR to an FGFR1-TKI. We molecularly scrutinized the resistant cells, using whole-exome sequencing, RNA sequencing and global DNA methylation analysis. Our results show a *de novo* activation of AKT and ERK, and a reactivation of mTOR. Furthermore, the resistant cells exhibited strong upregulation and activation of MET, indicating crosstalk between the FGFR1 and MET axes. The resistant cells also underwent a global decrease in promoter hypermethylation of the CpG islands. Finally, we observed clonal expansion of a pre-existing change in *AKT1*, leading to S266L substitution, within the kinase domain of AKT. Our results demonstrate that AR to FGFR1-TKI involves deep molecular changes that promote the activation of MET and AKT, coupled with common gene expression and DNA methylation profiles. The expansion of a substitution at *AKT1* was the only shared genetic change, and this may have contributed to the AR.

## INTRODUCTION

Our knowledge of the genetic and molecular basis of cancer cells is being enthusiastically applied to the design of novel anticancer drugs. In some cases, this is giving rise to new protocols for therapeutic management that are steadily replacing traditional treatments. Lung cancer treatment is at the forefront among solid tumors for using targeted therapeutics, particularly tyrosine kinase inhibitors (TKIs). Since erlotinib began to be used to treat lung cancer patients with *EGFR* mutations [1–2], about a decade ago, many new drugs have been designed to treat lung cancer patients with activating alterations at growth factor receptors with tyrosine kinase activity. These include TKIs for tumors carrying mutations at *EGFR*, *ERBB2* and *MET,* fusions at *ALK*, *ROS* and *RET*, and strong focal amplification at the *MET*, *ERBB2*, *FGFR1* and *PDGFRA* genes [1–8].

However, despite the clinical benefits of the targeted therapeutics, chronic exposure to the drug inevitably triggers the acquisition of resistance. This secondary refractoriness typically occurs as a result of the accumulation of novel genetic alterations in the kinase target, in other receptor tyrosine kinases (RTKs), or in molecules acting downstream of these RTKs [9]. The acquired genetic alterations can originate *de novo* or as clonal expansions of pre-existing low-abundance clones in the tumor. The best studied mechanisms for acquired resistance (AR) to TKIs in lung cancer are those associated with anti-EGFR and anti-ALK treatments. In the case of EGFR, AR mainly arises due to the p.T790M mutation at *EGFR*, or to the activation of other RTKs such as *MET,* AXL and *ERBB2* [9–13], whereas point mutations or amplification at *ALK* or *KIT*, among others, are the main mechanisms that trigger AR to the ALK-TKI [14–16]. Secondary resistance to TKIs against other RTKs is less well understood. Amplification and overexpression of wild-type *KRAS,* activation of the BRAF and of the HER pathways are among the mechanisms proposed by which AR to MET-TKIs might arise in various types of cancer [17–19]. Our research, reported here, using isogenic pairs of drug-sensitive and drug-resistant human cancer cell lines, reveals that inactivation of *NF2* is one of the potential mechanisms associated with AR to MET-TKI [20].

Whereas most of the alterations leading to the activation of an RTK are found in the lung adenocarcinoma subtype, FGFR1 is one of the few receptors known to be genetically activated in lung squamous cell carcinomas [6, 21–22]. Robust responses to FGFR inhibition are seen only in high-level *FGFR1*-amplified cancers [6, 23]. Although the therapeutic benefits of FGFR1 inhibition in most FGFR1-activated lung cancers are well established, the mechanisms that cause AR remain largely unknown. It has recently been suggested that activation of MET or AKT underlies AR to FGRF1-TKIs [24–25]. Here, we used the lung cancer cells DMS114, which carry genetic activation of FGFR1, as a cancer cell model to investigate AR to FGFR1-TKIs. We obtained various clones from the DMS114 parental cells that had become refractory to the inhibitor. We used whole-exome sequencing (WES), RNA sequencing and global DNA methylation microarrays, to search for the genetic and molecular alterations underlying drug resistance in these cell sub-populations.

## RESULTS

### Generation of AR to FGFR1 inhibitors from the parental DMS114 cells involves *de novo* activation of AKT and ERK

**Figure 1:**
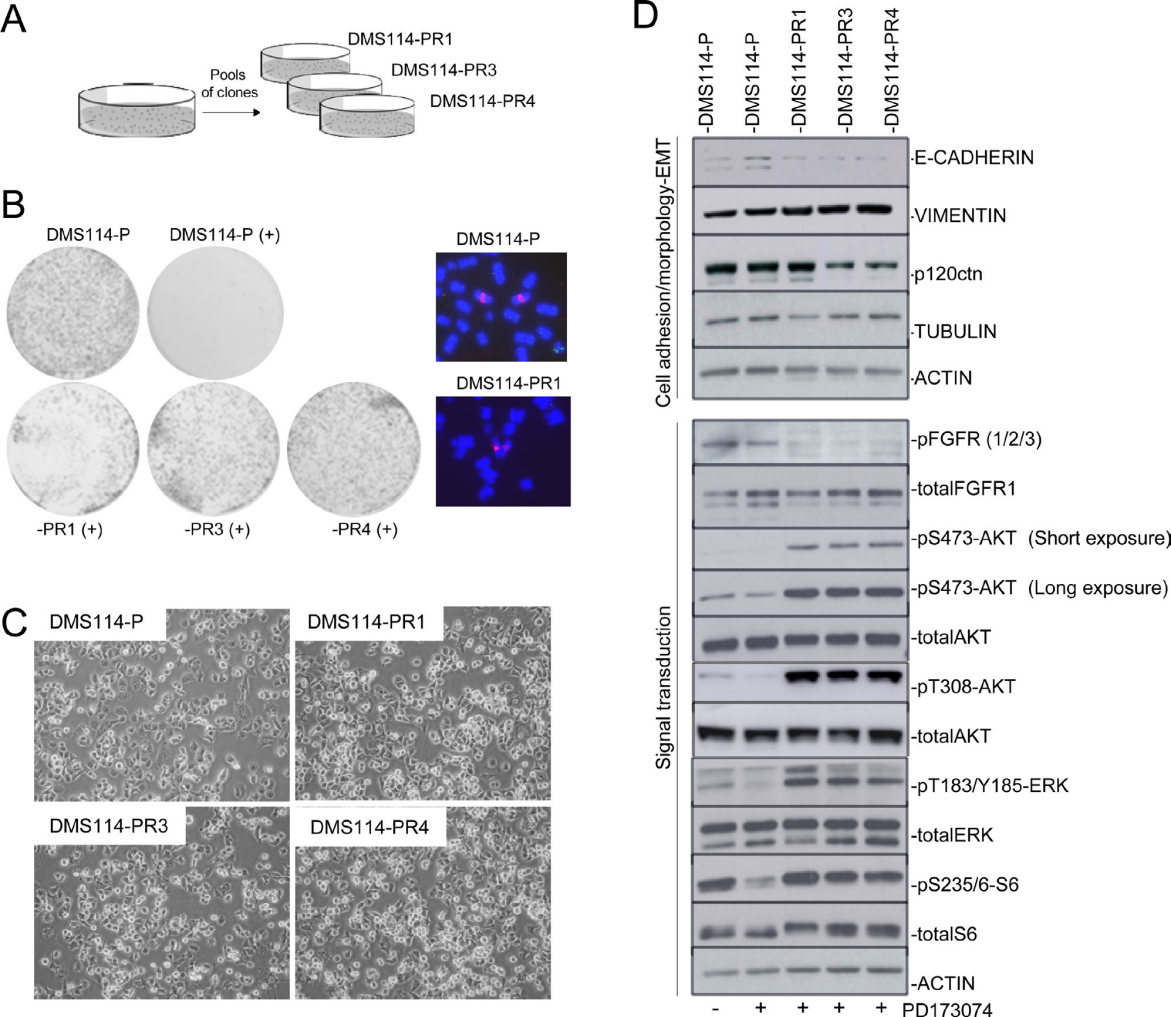
Generation of AR to FGFR1 inhibitors involves *de novo* activation of AKT and ERK (**A**) Description of the DMS114-R cells generated. (**B**) Left panel: Colony formation assay for cell-growth inhibition upon administering PD173074 treatment to the DMS114 parental (DMS114-P) and to the different DMS114-R cells. Right panel: Examples of metaphase nuclei from DMS114-P cells and the indicated resistant cells at the *FGFR1* gene (probes in red). Control probe in green. (**C**) Phase contrast images showing the cell morphology of the indicated DMS114 cells. (**D**) Western blot of the indicated proteins in DMS114-P and DMS114-R cells. In the case of DMS114-P, extracts with (+) and without (−) treatment with the PD173074 inhibitor (1 µM) are shown. The upper panels indicate the levels of different proteins related to cell adhesion and morphology. The lower panels show the phosphorylation levels of proteins involved in signal transduction pathways. ACTIN, total protein loading controls.

The DMS114 cell line endures high levels of *FGFR1* gene amplification and is sensitive to FGFR1 inhibition [6]. In order to generate AR to the FGFR1 inhibitor PD173074 [26], we subjected the parental DMS114 cell line (henceforth DMS114-P) to gradually increasing concentrations of the inhibitor, as indicated in the Materials and Methods. After several weeks, we obtained four different resistant pools of clones (named DMS114-PR1,-PR2, -PR3 and -PR4). Only three of them survived (DMS114-PR1, -PR3 and -PR4) and were then characterized morphologically and molecularly (Figure 1A–1B).

FISH analysis of *FGFR1* indicated that the cells had not suffered any apparent modifications in the levels of gene amplification (Figure 1B). The morphology of the DMS114-P and of the resistant (henceforth DMS114-R) cells was very similar (Figure 1C). We determined the levels of cell structure-related proteins and observed a slight increase of vimentine in all the DMS114-R cells and a decrease in the amount of p120ctn in the -PR3 and -PR4 cells (Figure 1D). To ascertain the changes in activation/inactivation of signal transduction molecules in the AR to PD173074, we tested for phosphorylation at AKT, ERK and at the downstream target of mTOR, S6. In all the DMS114-R cells there was a *de novo* activation of AKT and ERK, and a reactivation of mTOR (Figure 1D). These results indicate the action of mechanisms that have been acquired in all the DMS114-R and that allow the inhibitory effect of FGFR1-TKI to be bypassed.

### The gene expression profile of the DMS114-R cells suggests crosstalk between the MET and FGFR1 axes

**Figure 2:**
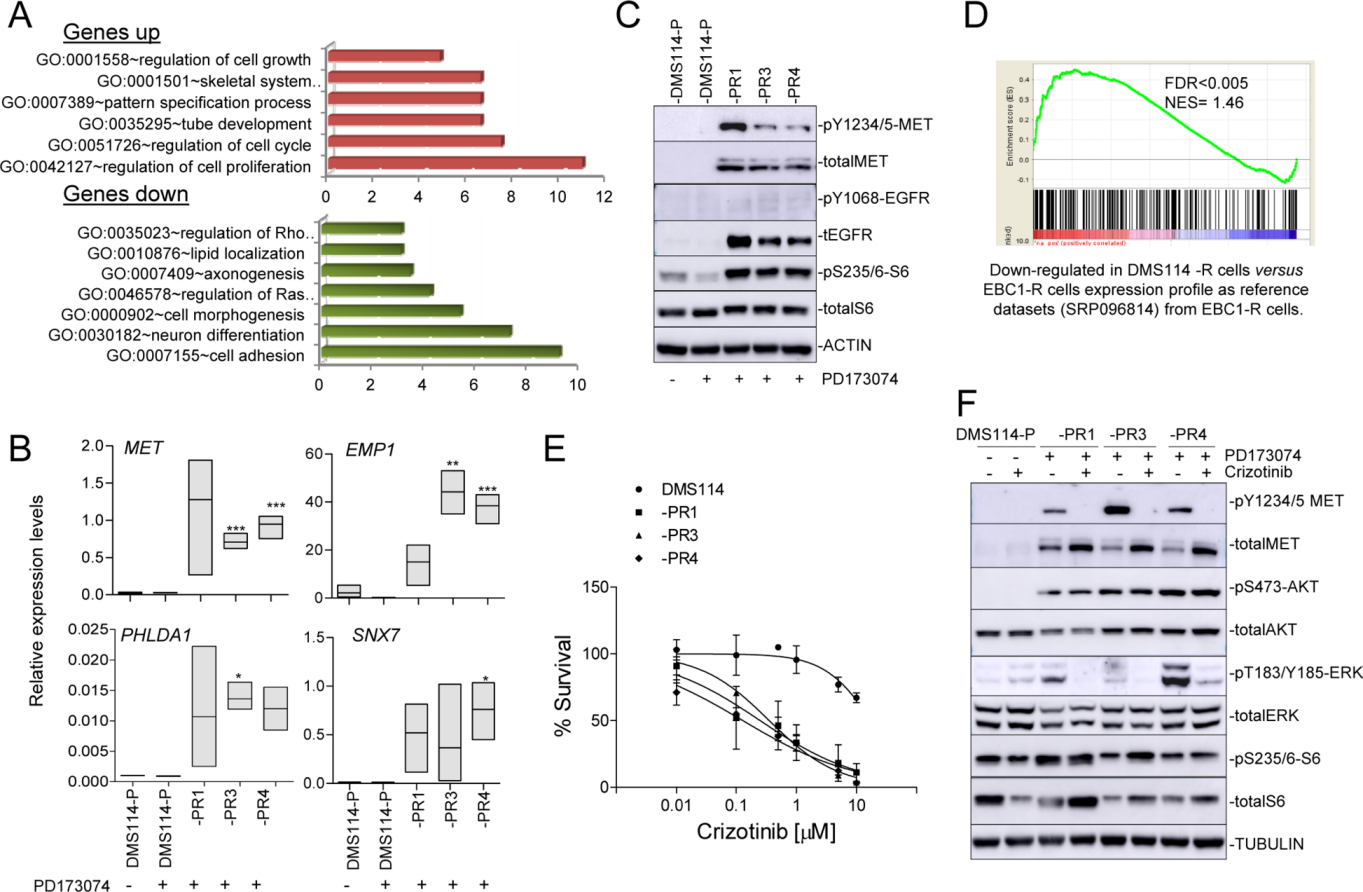
DMS114-R cells show specific gene expression profiles and MET activation (**A**) Enriched gene ontology (GO) classifications (*P <* 0.05 for all categories shown) among genes upregulated and downregulated in the gene expression profile of DMS114-R cells relative to DSM114-P cells (from Supplementary Table 2). (**B**) mRNA levels of indicated genes, relative to *ACTB,* in the indicated DMS114 cells. Error bars, SD of three replicates. ^*^P < 0.05; ^**^P < 0.005; ^***^P < 0.001. (**C**) Western blot of the indicated proteins in the DMS114 cells. In the case of DMS114-P, extracts with (+) and without (−) treatment with the PD173074 inhibitor (1 µM) are shown. ACTIN, total protein loading controls. (**D**) Graph of the ranked gene lists derived from comparison (using GSEA) of the indicated datasets and gene lists. Probabilities and false-discovery rates (FDRs) are indicated. (**E**) Cell viability of the DMS114 parental (DMS114-P) and DMS114 resistant (DMS114-R) cells, measured using MTT assays, after treatment with increasing concentrations of crizotinib. Lines represent cell survival relative to untreated controls of the MTT assays in the cells treated with increasing concentrations of the inhibitor for 72 h. Error bars indicate the standard deviation. (**F**) Western blot of the indicated proteins in the DMS114 cells. TUBULIN, total protein loading controls. In the case of DMS114-P, extracts with (+) and without (−) treatment with PD173074 (1 μM) or crizotinib inhibitors (100 nM) are shown.

To further evaluate the molecular characteristics associated with AR, we performed RNA-seq of the DMS114-P, and DMS114-PR1, -PR3 and -PR4 cells. We measured the global gene expression levels and found that the DMS114-PR3 and -PR4 cells had more similar patterns of gene expression than the DMS114-PR1, which showed the greatest difference (Supplementary Figure 1). We then analyzed the gene ontology of the shared upregulated- and downregulated genes, which revealed the enrichment of various functions related to cell growth and development, such as genes coding for growth factor receptors or signal transduction molecules (e.g. *MET, EMP1, PHLDA1* and *SNX7*) (Supplementary Table 2; Figure 2A–2B). It was interesting to note that *MET* and *EGFR* were among these common genes, exhibiting approximately 30-fold and 10-fold upregulation, respectively, in all the DMS114-R cells. The steep increase in the levels of *MET* transcript in the DMS114-R cells was associated with the activation of the MET receptor, measured as the increase in levels of phosphorylated MET (pMET) (Figure 2C). However, in the case of EGFR, there was an increase in the total protein levels, but without phosphorylation at the Y1048 residue, indicating that the increase in EGFR levels in these cells is not associated with its constitutive activation. Other genes, such as *ABI3BP*, were greatly reduced in the DMS114-R cells (Supplementary Table 2). Interestingly, we had previously reported that *ABI3BP* was strongly upregulated in cells that had AR to the MET-TKI [20]. Taking this into account, we carried out GSEA to compare the gene expression profiles. Consistent with the observed *de novo* activation of MET in the DMS114-R cells, there was a significant negative correlation between the expression pattern of cells with AR to the MET-TKIs and the gene expression profiles of the DMS114-R cells (Figure 2D).

### Cell growth and activation of AKT in the DMS114-R cells is uncoupled from MET activity

The results reported above suggest that activation of the MET pathway and crosstalk between the MET and FGFR1 axes contributes to the AR to FGFR1-TKI. To test this hypothesis we determined the sensitivity to crizotinib, a well established MET-TKI. We noted a greater sensitivity to cell growth inhibition of the DMS114-R cells after crizotinib treatment compared with the parental cells (from not being achieved in the parental cells, to <1 μM in all the DMS114-R cells) (Figure 2E). Conversely, it is well known that inhibition of MET activity in *MET*-amplified lung cancer cells triggers a profound reduction of phosphoAKT (pAKT), indicating that AKT is a downstream effector of MET [20]. Here we observed that, in the DMS114-R cells, crizotinib strongly inhibited pMET but did not affect the levels of pAKT or of the ribosomal protein S6, an indirect target of mTOR (Figure 2F). However, crizotinib did reduce the levels of phophoERK (pERK), which may explain the partial effects of crizotinib on inhibiting the growth of DMS114-R cells.

### Genomic screening identifies the clonal expansion of various nucleotide substitutions, including an *AKT1* mutation

**Figure 3:**
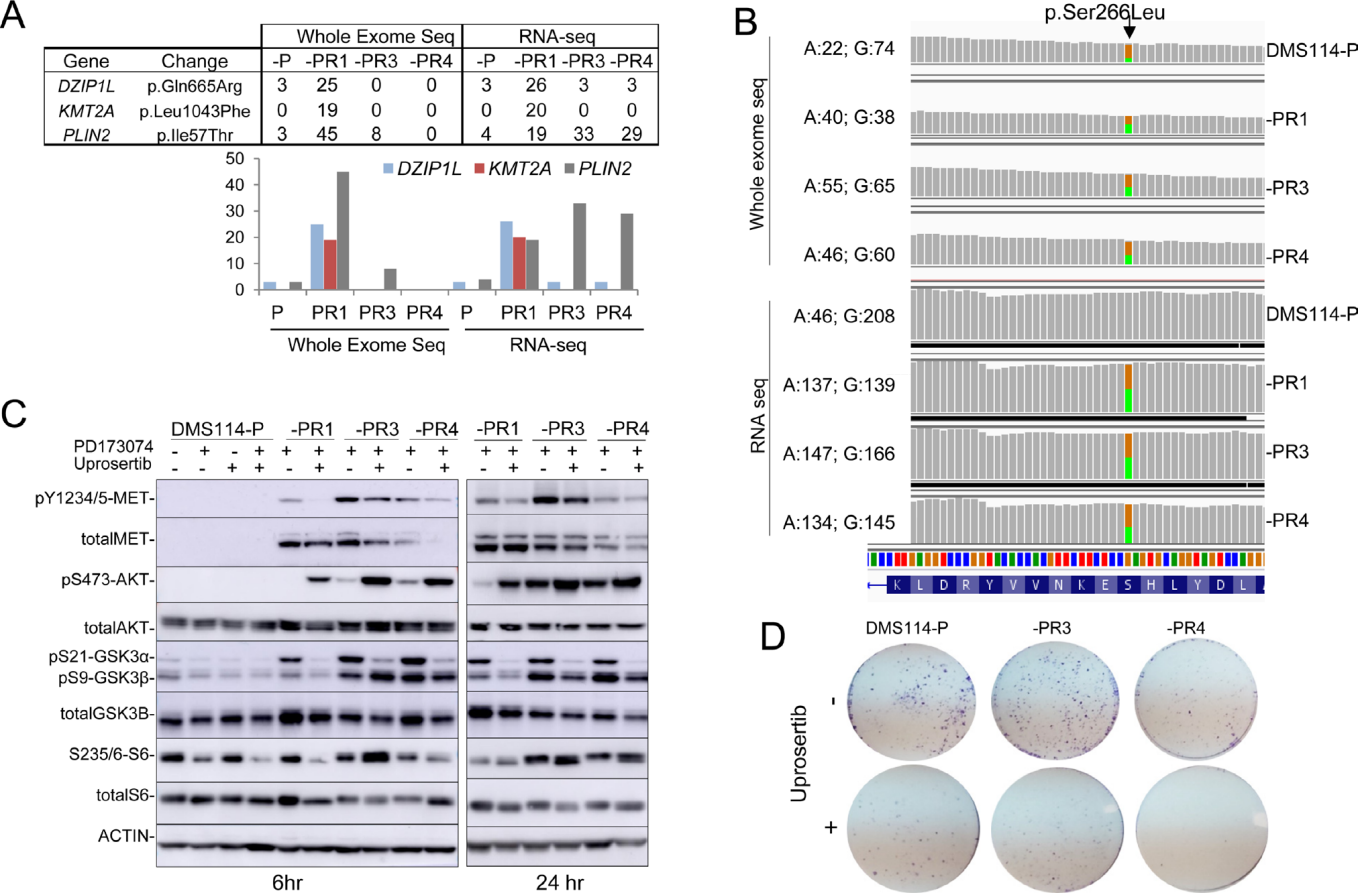
Genomic screening of DMS114-P and DMS114-R cells (**A**) Table (upper panel) and bar graph (lower panel) showing the percentage of mutated reads, at each indicated gene, for each variant, relative to the total number of reads found at the whole exome and mRNA sequencing (RNA-seq) analysis in the indicated cells. (**B**) The G > A mutation at *AKT1* (encoding a S266L alteration) detected by whole-exome and RNA sequencing of the DMS114-P and DMS114-R cells. Green and orange indicate the A and G bases, respectively. The number of reads for each base in each cell is indicated, on the right. (**C**) Western blots of the indicated proteins in the DMS114-P and DMS114-R cells treated with (+) and without (−) the AKT inhibitor, uprosertib (200 nM) for 6 h (left panel) or 24 h (right panel). In the case of DMS114-P, extracts with (+) and without (−) treatment with PD173074 (1 µM) are also shown. (**D**) Representative example of the colony formation assay for DMS114-P and DMS114-R cells after treatment with the AKT inhibitor, uprosertib (500 nM).

To determine whether the AR involves modifications at the DNA level, we decided to perform WES. The exome coverage is reported in Supplementary Table 3. Some changes in copy number and gene fusions, but not strong focal gene amplification (GA) or homozygous deletions (HD), were detected in the DMS114-R cells (Supplementary Tables 4 and 5). We searched for variants that occurred at >10% allele frequency in at least one of the DMS114-R cells but present no more than negligible numbers of parental cells (Supplementary Table 6). Further, among those, we selected the variants with allele frequencies >20% at the mRNA level and four aminoacid substitutions in three genes (*DZIP1L, KMT2A* and *PLIN2*) were left (Figure 3A): *DZIP1L,* coding for the DAZ interacting zinc finger protein 1-like; *KMT2A,* for the lysine methyltransferase 2A, and *PLIN2* for the perilipin 2 protein. All these changes were predicted to be of moderate effect (Supplementary Table 6). It is known that many genetic alterations found in cells that have AR to a given TKI are clonal expansions from low-frequency mutations, already present in untreated specimens [20, 27]. After manually searching for the *DZIP1L, KMT2A* and *PLIN2* mutations in all the DMS114 cells, we found that changes in *DZIP1L* and *PLIN2* were already present in the parental cells, at frequencies of <5% (Figure 3A). The analysis of the RNA-seq data yielded similar results, except for the aminoacid substitution at *PLIN2*, which was not identified by WES analysis in the -PR4 cells, whereas its abundance at mRNA levels increased by as much as almost 30%. This was the only change in common to all the resistant cells at the mRNA level, at least at a frequency of >10%. These findings suggest that the changes originated from the expansion of sub-clonal populations of the parental cells.

Given the activation of AKT observed in all the DMS114-R cells (Figure 1D and Figure 2F), we decided to manually inspect the bam files in the Integrative Genomics Viewer (IGV). We searched for genomic changes at various RTKs and signal transduction factors that may have gone undetected in the bioinformatic analysis. We detected two variants in *AKT1*: an aminoacid change (S266L), already reported in genomic databases (www.cbioportal.org), and a known polymorphism (R242R, rs1130233). Both variants were allocated in different alleles and, since wild type genotypes for both variants were also present, it can be concluded that S266L is a tumor-specific alteration (Supplementary Figure 2). The S266L change was present in only 20% of the reads of the DMS114-P cells and became enriched by up to 50% in the DMS114-R cells at the genomic and RNA levels (Figure 3B). This suggests that the DMS114-R cells have arisen from the clonal expansion of AKT-mutant cells. Although the effect of this alteration on the constitutive activation of AKT is unknown, it is interesting to note that the change is located in the kinase domain of AKT. Moreover, the observation that about 20% of DMS114-P cells carry this mutation may explain the low levels of pAKT in the DMS114-P cells, which are not inhibited by treatment with the FGFR1-TKI (Figure 3D).

Given these findings, we tested the effects of uprosertib, an ATP-competitive AKT inhibitor, on the different DMS114 cells. The inhibition of AKT kinase activity by uprosertib was demonstrated by the strong reduction in the phosphorylation levels of its direct targets, GSK3α (at 6 and 24 hr after the treatment) and GSK3β (more evident at 24 hr) (Figure 3C). A previously reported reactive effect [25] was responsible for an increase in pAKT levels. The levels of pMET and pS6 fell in the -PR1 cells, whereas the levels of total MET were slightly reduced in the -PR3 and -PR4 cells (at 6 hr). Uprosertib lead to a decrease in the growth but in all the DMS114 cells, indicating that the drug is exerting some inhibitory functions outside AKT. Taken together, these observations do not discard that AKT activation may influence the AR to the FGFR1-TKI.

### Epigenomic profiling reveals a significant decrease in gene promoter hypermethylation in DMS114-R cells

**Figure 4:**
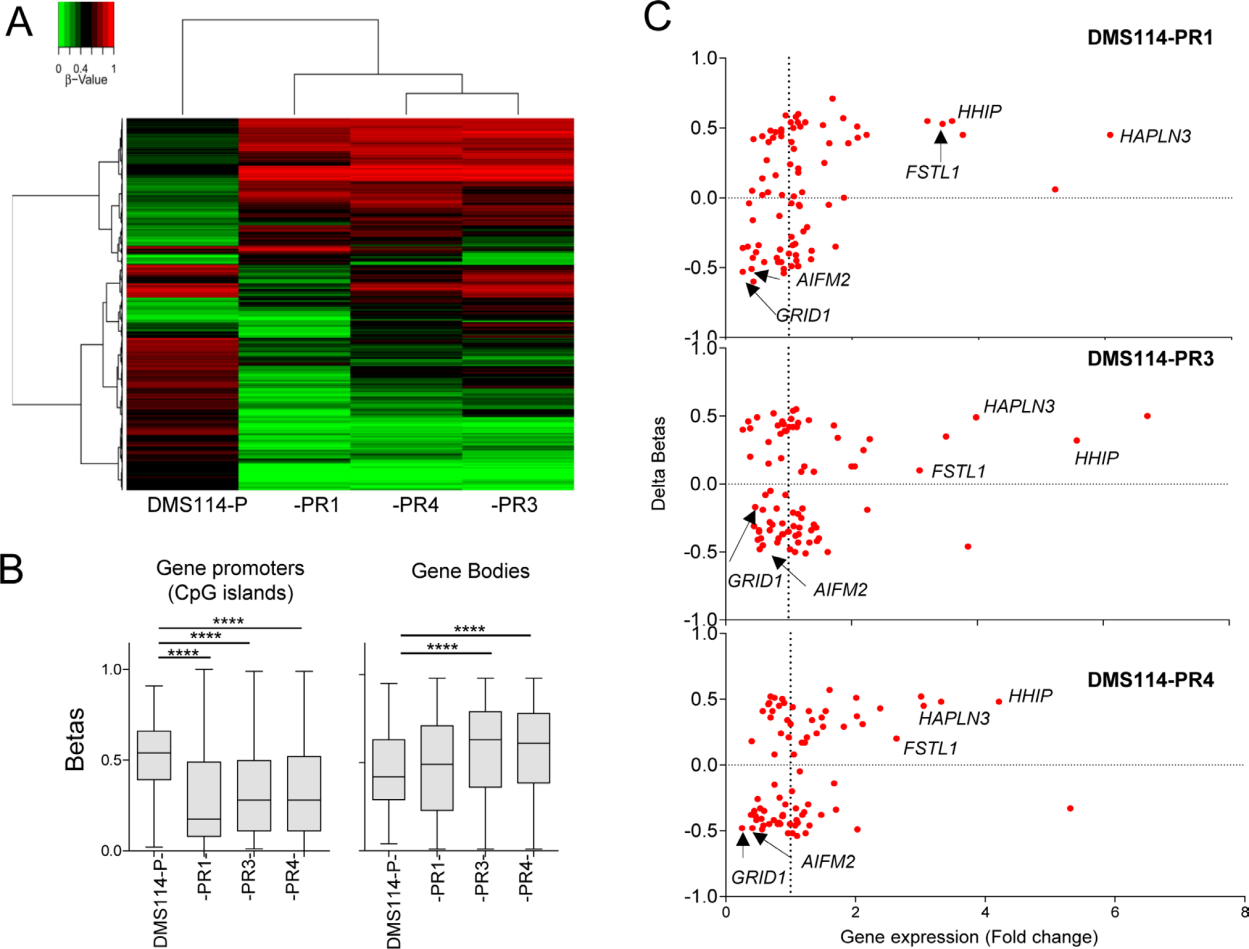
Genome-wide methylation analysis (**A**) Unsupervised hierarchical clustering in the indicated parental DMS114 cells (DMS114-P) and DMS114-R cells for the 5,000 most variable CpGs (from Supplementary Table 7). The heatmap colors illustrate beta values representing the degree of methylation, from low (green) to high (red), as shown by the scale at the top-left of the Figure. (**B**) Box-plots showing the beta values of the CpGs at the promoters (only CpG islands) or bodies of indicated cells, from Supplementary Table 7. Promoter regions were defined as the upstream 2000 bp and downstream 500 bp from the transcription start site (TSS) of each gene. Two-tailed unpaired Student’s *t* tests of the beta values of DMS114-P versus each group of DMS114-R cells. ^****^P < 0.0001. (**C**) Scatter-plot showing the mean change in promoter DNA methylation (delta betas) at the CpG islands (Y-axis) and the mRNA expression level of each gene, measured as RPKM (reads per kb per million reads) (X-axis). In general, the gene expression level was negatively correlated with DNA methylation level at the promoter regions.

Finally, we performed genome-wide DNA methylation profiling in all the DMS114 cells [28] to determine whether AR involves modifications in DNA methylation. The 5,000 CpGs with the most variable methylation levels were plotted in an unsupervised manner (Figure 4A and Supplementary Table 7). Two clusters were generated that segregated the various resistant cells, -PR1, -PR3 and -PR4, from the parental cells (DMS114-P). Among the cluster containing the resistant cells, the -PR3 and -PR4 cells also clustered together and segregated from the -PR1 cells, consistently with the similarities in the gene expression profiles. Interestingly, all the DMS114-R cells showed significantly less promoter hypermethylation of the CpG islands than did their parental counterparts, and a moderate increase in methylation of the gene bodies (Figure 4B). Some genes showed a strong association between changes in promoter hypermethylation and changes in gene expression (Figure 4C). Among the genes with the strongest gene promoter hypermethylation and concomitant increase in gene expression were: *HHIP*, which encodes a member of the hedgehog-interacting protein family; *HAPLN3*, which codes for a protein in the hyaluronan and proteoglycan binding family; and *FSTL1*, which encodes an activin-binding protein. Genes with strong promoter hypermethylation associated with reduced gene expression included the glutamate receptor *GRID1* and *AIFM2*, which encodes a flavoprotein oxidoreductase (Figure 4C).

## DISCUSSION

After generating lung cancer cells that have acquired refractoriness to the FGFR-TKI, we found no morphological transformations in the resistant cells. This finding differs markedly from the changes in cell appearance associated with AR to the TKIs against various growth factor receptors that have previously been reported, such as shifts from NSCLC to SCLC, epithelial-to-mesenchymal transformation, among other changes [20, 27–29]. We observed strong molecular shifts that were common to all the resistant cells, such as the reactivation of pS6, which indicated that the cells had bypassed the inhibitory effect of the drug upon activation of mTOR. Likewise, there was a *de novo* activation of MET, AKT and ERK. The DSM114-R cells also displayed a common gene expression profile that was inversely correlated with that of a lung cancer cell line that has AR to a MET-TKI [20]. A functional connection between the MET and FGFR1 pathways is supported by the finding that FGFR inhibitions sensitized *MET-*amplified gastric cancer cells to treatment with an anti-MET antibody and by the reactivation of MET that drove MAPK activity as a consequence of AR to the FGFR1 inhibitor [30, 31]. Although several observations point towards reactivation of MET in FGFR1-TKI-resistant cells, it is not fully understood how this reactivation occurs. Here, we found that inhibition of MET in these cells reduces the levels of pERK but not those of pAKT. These partial effects on the blockade of signal transduction may explain the moderate effects of the inhibition of MET on cell growth suppression. It also suggests that, although MET activation certainly contributes, other mechanisms are responsible for the AR to the FGFR1-TKI in these cells.

We found a tumor-specific aminoacid substitution at *AKT1* (S266L) in all the DMS114-R cells that arose from pre-existing clones in the parental cells. This aminoacid substitution is located in the kinase domain of AKT and we cannot rule out the possibility that it affects its conformation, making it more accessible for phosphorylation by PDPK1 (at T308) and by mTORC2 (at S473) [32]. The low levels of constitutive phosphorylation of AKT in the DMS114-P cells, which are consistent with the presence at low abundance of the p.S266L change in these cells, is intriguing. The inhibition of AKT in FGFR1-TKI-resistant cells has recently been shown to be able to restore sensitivity to the FGFR1-TKI, evidencing the dependency of these cells on AKT [25]. However, in our hands, inhibition of AKT kinase activity impeded cell growth not only in the resistant but also in the parental cells, which did not allow us to definitive conclude that AKT inhibition is selectively impairing the growth of the DMS114-R cells. Future work, *using in vivo* models, will be necessary to ascertain the validity of these observations. The effects of this aminoacid substitution in the DMS114R cells need to be exhaustively evaluated to definitively disprove a role for it in AR to the FGFR1-TKI.

Finally, DNA methylation profiling was able to discriminate DMS114-P from DMS114-R cells, and confirmed the close similarity between the DMS114-PR3 and -PR4 compared with the -PR1 cells, as was also found for the gene expression profiles. Among the most interesting differences is the overall decrease in DNA methylation of the CpG islands in gene promoters. Changes in the expression of some genes were associated with changes in gene promoter hypermethylation.

Collectively, our results show molecularly different clones arising during AR to the FGFR1-TKI, with common patterns of gene expression and global DNA methylation. The resistant clones showed activation of AKT and MET and the clonal expansion of an *AKT1* mutation, S266L, in all the DMS114-R cells that may contribute to the AR to FGFR1-TKI.

## MATERIALS AND METHODS

### Lung cancer cell lines

The DMS114 cell line was obtained from the American Type Culture Collection (ATCC, Rockville, MD, USA), grown under recommended conditions, and maintained at 37° C in a humidified atmosphere of 5% CO_2_. All the cells and clones tested negative for mycoplasma infection. The identity of the lung cancer cell lines, the parental and each of the resistant pools, was verified by determining the presence of the *TP53* mutation and amplification of the *FGFR1* gene [8]. Genomic DNA and total RNA were extracted by standard protocols.

### Fluorescence *in situ* hybridization (FISH) analysis

To determine gene copy number at the *FGFR1* gene, we performed dual-color FISH analysis, as previously described [22], in interphase nuclei using a BAC clone, labeled in Spectrum Red for the FGFR1 gene (RP11-350N15), and control BAC (RP11-213K6), located on the same arm and labeled in Spectrum Green.

### Generation of AR to PD173074, treatments, cell viability and growth assays

To generate AR to the FGFR1 inhibitor, PD173074 (Ref. 2499, Sigma-Aldrich, St Louis, MO, USA) [26], sensitive DMS114 cells were cultured in a medium with a gradually increasing concentration of the drug (from a starting concentration of 50 or 150 nmol/L to a final concentration of 1 μmol/L) for three/four months. Individual cells capable of proliferation were allowed to grow and were selected as pools of clones, transferred to separate plates and treated with 1 μmol/L of PD173074 thereafter.

For the colony formation and cell-survival assays, cells were allowed to recover for 24 h before administering the treatments of uprosertib (GSK2141795, MedChemExpress, Monmouth Junction, NJ, USA), or crizotinib (Ref. 4368, Tocris Bioscience, Bristol, UK). For the cell-survival assays, cells were seeded at a density of 10,000–15,000 cells/well on 96-well plates and exposed to various concentrations of each drug for 72–96 h, before measuring the viable cell number by MTT assay. Briefly, 10 µl of a solution of 5 mg/ml MTT [3-(4,5)-dimethylthiazol-2-yl)-2,5-diphenyltetrazolium bromide] (Sigma Chemical Co., Zwijndrecht, The Netherlands) was added to each well. After incubation for 3 h at 37° C, the formed formazan was dissolved with 100 µl of lysis buffer and absorbance was determined at 596 nm (Bio-Rad, Hercules, CA, USA). Viabilities were expressed as a percentage of the untreated controls. The 50% growth inhibition (IC_50_) was determined from the dose-response curve. Results are presented as the median of at least three biological experiments for each cell line and each compound. For colony formation assays, cells were seeded at a density of 10,000–15,000 cells/well on 6-well plates in Waymouth’s media containing 10% (v/v) fetal bovine serum. Fourteen or 15 days lafter treatment, cells were stained with Crystal Violet (Merck Millipore, Darmstadt, Germany).

### Antibodies and western blots

The details of the antibodies used are presented in Supplementary Table 1. The protocol for western blots was performed as described elsewhere [20]. Briefly, cells were plated in 6-well plates at a density of 0.4 × 10^6^ cells per well, then lysed and supplemented with protease and phosphatase inhibitors. Supernatants were resolved on an SDS-PAGE gel, transferred onto nitrocellulose membranes (Amersham, GE Healthcare Life Science), and probed with the indicated antibodies. Detection was done using a chemiluminescence system (Millipore).

### Quantitative real-time PCR (qRT-PCR)

Total RNA was extracted with PureLink RNA Mini Kit (Ambion, Life Technologies, Foster City, CA, USA) and reverse-transcribed with SuperScript II Reverse Transcriptase (Thermo Fisher, Scientific Waltham, MA, USA) and Random Primers (Promega, Madison, WI, USA), according to the manufacturers’ instructions. Quantitative PCR was performed in an Applied Biosystems 7900HT Fast Real-Time PCR System using SYBR Green PCR Master Mix (Applied Biosystems, Life Technologies, Warrington, UK). Three biological replicates were carried out. We used the human *ACTB* as a control to assess inter-sample variation. Reactions were carried out in triplicate. Primer sequences are presented in Supplementary Table 1.

### Whole-exome sequencing and RNA sequencing

For DNA extraction, the cells were collected and placed in 1% SDS/proteinase K (10 mg/ml) at 58° C overnight. Digested tissue was then subjected to phenol-chloroform extraction and ethanol precipitation following standard protocols. DNA quality was assessed by visualization in agarose gel and quantified with NanoDrop and PicoGreen. For RNA sequencing (RNA-seq), total RNAs were extracted using a TRIzol^®^ Plus RNA Purification Kit (Purelink RNA Mini Kit, Ambion). RNA integrity (RIN) and quality control were assessed with the Agilent 2100 Bioanalyzer and quantified with a Nanodrop Spectrophotometer.

The WES and RNA-seq were carried out at the Spanish National Genome Analysis Center (CNAG-CRG, Barcelona, Spain). The sureSelect kit (Agilent Technologies, Santa Clara, CA, USA) was used for exome capture, and the TruSeq RNA Library Preparation Kits (Illumina, San Diego, CA, USA) was used for RNA library preparation. The products were then deep-sequenced in an HiSeq 2000 Illumina Analyzer (Illumina) using the paired-end 2 × 100 bp read option for the WES and the 2 × 75 read option for the RNA-seq. Data were analyzed at the CNAG-CRG, as previously described [20]. Briefly, for RNA-seq, reads were mapped against the human reference genome (hg19), with the GEM RNA-seq pipeline (http://gemtools.github.io/). Genes with >2-fold or <0.5-fold change and an absolute difference of >5 cpm were considered differentially expressed. The lists of upregulated and downregulated genes were functionally classified according to the Gene Ontology (GO) database. In the WES data analysis, the reference genome was mapped using the GEM toolkit version 1, allowing up to four mismatches (Supplementary Methods).

We merged the WES copy number data with the gene expression data from the RNA-seq to select regions with GA and HD. These regions had to feature overexpression of GA (defined as a level of expression at least 10 times that of a given gene in the cells carrying GA compared with the mean level of expression of cells without GA), or lack expression of HD, as previously described [20, 33].

### Global methylation microarray analysis

We used previously described protocols for global methylation microarray analysis [28]. For DNA methylation microarrays, all DNA samples were assessed for integrity, quantity and purity by electrophoresis in a 1.3% agarose gel followed by PicoGreen quantification. 500 ng of genomic DNA was bisulfite-converted using an EZ DNA methylation kit (Zymo Research, Irvine, CA, USA). We used 200 ng of the product for hybridization on the MethylationEPIC BeadChip (Illumina) (Supplementary Methods). The microarray methylation data are available from the Gene Expression Omnibus (GEO) under accession code GSE115777.

### Statistical analysis

Group differences in categorical variables were compared with the chi-square test. Group differences in continuous variables were compared with the two-tailed unpaired Student’s *t* test, ANOVA, or the Mann–Whitney *U* test. All statistical analysis was performed with Prism software (GraphPad Software, San Diego, CA, USA). Values of *P* < 0.05 were considered statistically significant. Gene Set Enrichment Analysis (GSEA) of the ranked list was undertaken using the SRP096814 gene expression signature (Supplementary Table 2) as a gene set.

## SUPPLEMENTARY MATERIALS FIGURES AND TABLES








