# Supplementary material for: Deep analysis of acquired resistance to FGFR1 inhibitor identifies MET and AKT activation and an expansion of AKT1 mutant cells
Source: Oncotarget. 2018 Jul 31;9(59):31549–58. doi: 10.18632/oncotarget.25862 (PMC6101141; doi:10.18632/oncotarget.25862)
Supplement: Supplementary file 1 [file oncotarget-09-31549-s001.pdf]

Deep analysis of acquired resistance to FGFR1 inhibitor identifies MET and AKT activation and an expansion of *AKT1* mutant cells

SUPPLEMENTARY MATERIALS

MATERIALS AND METHODS

Whole-exome and RNA sequencing data analysis for identifying variants specific to resistant cells

For RNA-sequencing (RNA-seq), reads were mapped to the human reference genome (hg19), with the GEM RNA-seq pipeline (http://gemtools.github.io/). Raw variant calls were manually checked with the Integrative Genomics Viewer (IGV; Broad Institute, Cambridge, MA). Gene quantification was performed with Flux Capacitor (http://sammeth.net/confluence/display/FLUX/Home). The TMM method [1] was used for normalization. Similarity among samples was inspected with a multi-dimensional scaling (MDS) plot. Genes with a >|2|-fold change and absolute difference of >5 counts per million (cpm) were considered to be differentially expressed. The lists of upregulated and downregulated genes were functionally classified according to the Gene Ontology (GO) database. For RNA-seq, variant calling was performed with SAMtools [2] applying the same parameters as for WES. Variants were manually checked with the IGV [3]. For fusion, transcripts were found by applying TopHat-Fusion [4] with default settings.

In the WES data analysis, the reference genome was mapped using the GEM toolkit version 1, allowing up to four mismatches. Unmapped reads were then aligned using BFAST with less stringent settings. Alignment files (BAM format) were processed using Picard tools (http://broadinstitute.github.io/picard/) and the Genome Analysis Tool Kit (GATK). For variant calling of single nucleotide variants (SNVs) and short insertions and deletions (indels), we used SAMtools (version 0.1.19) with default settings. Variants were annotated with dbSNP (version 137) and dbNSFP (version 1.3 light). Further, functional annotations and variant effect categories were added by snpEff [5]. For missense and in-frame indel-type mutations, only expressed genes were selected.

We merged the WES copy number data with the gene expression data from RNA-seq to select regions with gene amplification (GA) and homozygous deletion (HD). These regions had to feature overexpression of GA (defined as a level of expression at least 10 times that of a given gene in the cells carrying GA compared with the mean level of expression of cells without GA), or lack expression of HD, as previously described [6].

Global methylation microarray analysis

We used previously described protocols for global methylation microarray analysis [7]. For DNA methylation microarrays, all DNA samples were assessed for integrity, quantity and purity by electrophoresis in a 1.3% agarose gel and using PicoGreen quantification. 500 ng of genomic DNA was bisulfite-converted using an EZ DNA methylation kit (Zymo Research, Irvine, CA, USA). We used 200 ng of bisulfite-converted DNA for hybridization on the MethylationEPIC BeadChip (Illumina). Raw fluorescence intensity values were normalized with Illumina Genome Studio software (V2011.2) using 'control normalization' with background correction. Normalized intensities were then used to calculate DNA methylation levels (beta values). Likewise, values with low statistical power (indicated by detection values of $P > 0.01$) were excluded from the analysis. Genotyping probes present on the chip and DNA methylation probes overlapping with known single-nucleotide polymorphisms were also removed. Probes were considered to be in a promoter CpG island if they were located within a CpG island (UCSC database) and <2,000 bp away from a transcription start site (outside the X chromosome). Samples were clustered in an unsupervised manner using the 5,000 most variable values for CpG methylation according to the standard deviation for the CpG sites located in promoter regions by hierarchical clustering using the complete method for agglomerating the Manhattan distances.

REFERENCES

1. Robinson MD, Oshlack A. A scaling normalization method for differential expression analysis of RNA-seq data. Genome Biology. 2010; 11:R25.
2. Li H, Handsaker B, Wysoker A, Fennell T, Ruan J, Homer N, Marth G, Abecasis G, Durbin R; 1000 Genome Project Data

Processing Subgroup. The Sequence alignment/map (SAM) format and SAMtools. Bioinformatics. 2009; 25:2078–2079.

3. Robinson JT, Thorvaldsdóttir H, Winckler W, Guttman M, Lander ES, Getz G, Mesirov JP. Integrative genomics viewer. Nat Biotechnol. 2011; 29:24–6.

4. Kim D, Salzberg SL. TopHat-Fusion: an algorithm for discovery of novel fusion transcripts. Genome Biology. 2011; 12:R72.

5. Li H. A statistical framework for SNP calling, mutation discovery, association mapping and population genetical parameter estimation from sequencing data. Bioinformatics. 2011; 27:2987–2993.

6. Pereira C, Gimenez-Xavier P, Pros E, Pajares MJ, Moro M, Gomez A, Navarro A, Condom E, Moran S, Gomez-Lopez G, Graña O, Rubio-Camarillo M, Martinez-Martí A, et al. Genomic profiling of patient-derived xenografts for lung cancer identifies B2M inactivation impairing immunorecognition. Clin Cancer Res. 2017; 23:3203–3213.

7. Sandoval J, Heyn H, Moran S, Serra-Musach J, Pujana MA, Bibikova M. Validation of a DNA methylation microarray for 450,000 CpG sites in the human genome. Epigenetics. 2011; 6:692–702.

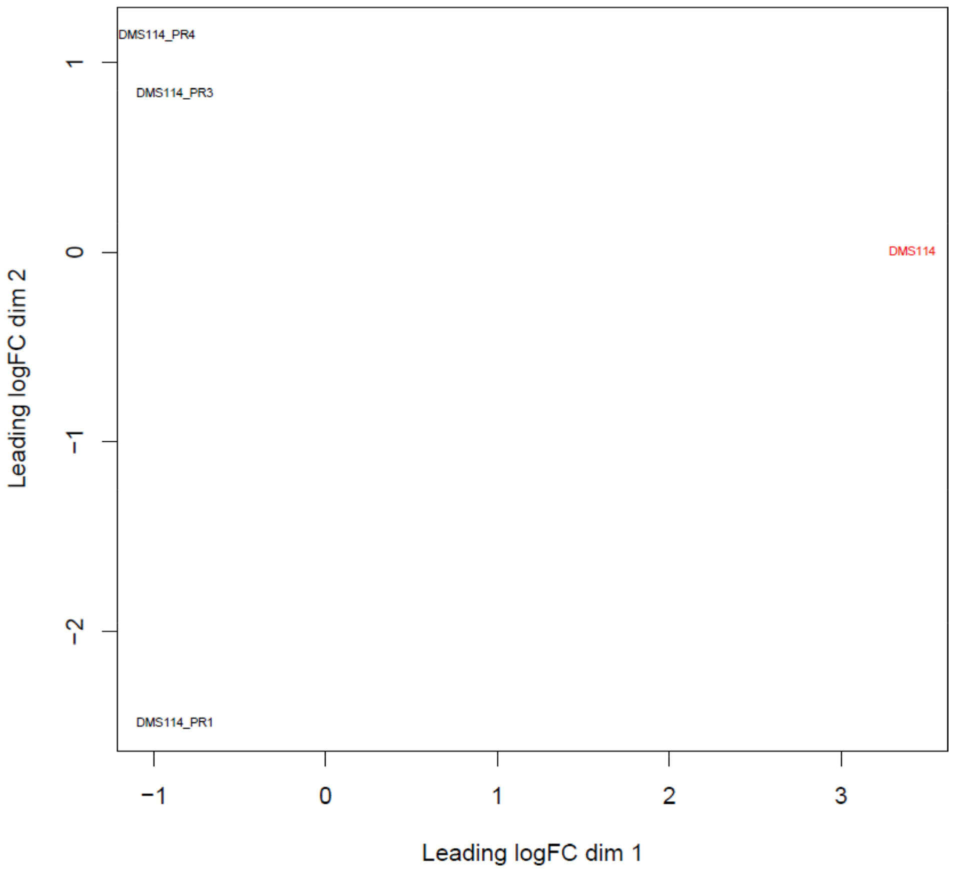

Supplementary Figure 1: Multidimensional scaling plot of DMS114-P and the various DMS114-R cells. dim = dimension; logFC = logarithm of n-fold change.

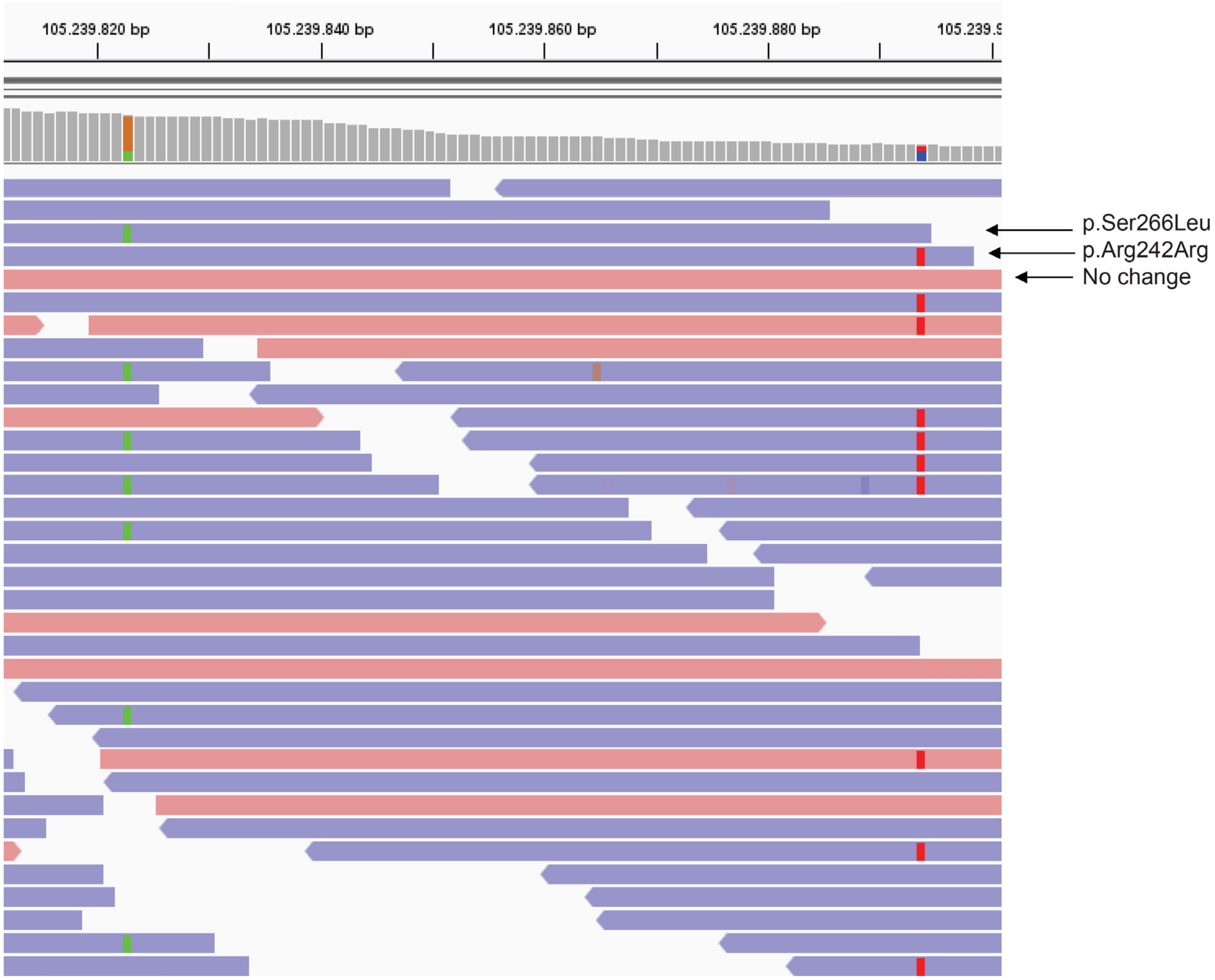

Supplementary Figure 2: The S266L mutation and the R242R single nucleotide polymorphism at the *AKT1* gene in the DMS114-P cells, detected by whole-exome sequencing. Red and blue bars indicate positive and negative strands, respectively.

Supplementary Table 1: List of antibodies used in western blots and primer pairs used for qRT-PCRs

Antibody	Dilution	Source
beta-TUBULIN (HRP) (ab21058)	1: 10000	Abcam (Cambridge, UK)
E-Cadherin (ab15148)	1: 1000	Abcam (Cambridge, UK)
pEGFR-Y1068 (ab32430)	1: 1000	Abcam (Cambridge, UK)
p120 (clone 98)	1: 1000	BD Transduction Lab. (San Diego, CA, USA)
AKT (#9272)	1: 1000	Cell Signaling (Danvers, MA, USA)
pAKT-S473 (#9271)	1: 1000	Cell Signaling (Danvers, MA, USA)
pAKT-T308 (#9275)	1: 1000	Cell Signaling (Danvers, MA, USA)
EGFR D38B1 (#4267)	1: 1000	Cell Signaling (Danvers, MA, USA)
ERK (#9102)	1: 1000	Cell Signaling (Danvers, MA, USA)
FGFR1 (D8E4, #9740),	1: 1000	Cell Signaling (Danvers, MA, USA)
MET (25H2) (#3127)	1: 1000	Cell Signaling (Danvers, MA, USA)
pMET-Y1234/Y1235 (#3126)	1: 1000	Cell Signaling (Danvers, MA, USA)
pS6-S235/236 (#4856)	1: 1000	Cell Signaling (Danvers, MA, USA)
S6 (#2217)	1: 1000	Cell Signaling (Danvers, MA, USA)
Vimentin (CBL-202K)	1: 1000	Merck Millipore
beta-ACTIN (HRP, A3854)	1: 10000	Sigma-Aldrich
pERK-T183/Y185 (clone MAPK-YT)	1: 1000	Sigma-Aldrich
pFGFR (Tyr653/654) (#3471)	1: 1000	Cell Signaling (Danvers, MA, USA)
GSK3B (ab93926)	1: 1000	Abcam (Cambridge, UK)
pGSK3B (S9) (#9323)	1: 500	Cell Signaling (Danvers, MA, USA)

Gene	Forward primer (5′-3′)	Reverse primer (5′-3′)
ABI3BP	AGTTCTTGCGTCCAAGTCCA	GGAGCAGGTCGCACAACTAT
ACTB	CAAGGCCAACCGCGAGAAGAT	CCAGAGGCGTACAGGGATAGCAC
MET	ATGATGAGGTGGACACACGA	CCATTGGACAAAGTGTGGACT
EMP1	AACATGTTGGTATTGCTGGCT	AGTTTTTCCAAAGACCTACTGAT
SNX7	AAATCTCAGGTATTCCAGGTCAC	ATGCAATTATTGGCACACAAA
PHLDA1	GTTGAGGAGTCTGTTGGGGG	CAGCCCCAAGAGGACTTTGTAT

Supplementary Table 2: List of genes upregulated and downregulated in DMS114-R relative to DMS114-P cells. See Supplementary_Table_2

Supplementary Table 3: Whole-exome sequencing depth and coverage report

Sample name	Million read-pairs	Yield (Gb)	Reference genome	% uniquely mapping	% unmapped	Mean coverage	Coverage at depth 10	Coverage at depth
DMS114	36.017	7.276	hsapiens. hs37d5	96.5	0.8	104.19	94.4	81.34
DMS114-PR1	34.734	7.016	hsapiens. hs37d5	96.5	0.77	100.51	94.23	80.8
DMS114 PR3	36.256	7.324	hsapiens. hs37d5	96.47	0.81	105.23	94.38	81.42
DMS114 PR4	36.949	7.464	hsapiens. hs37d5	96.41	0.84	103.13	95.22	82.45

Supplementary Table 4: Selection of regions and genes with increase or decrease in copy number

ID	Peak region	Genes within regions with increased copy number	MEAN FC
DMS114-PR1	chr11: 3051684-332126017	*DCDC1, DNAJC24, IMMP1L, ELP4, PAX6, RCN1*	2.4
DMS114-PR3	chr11: 3051684-332126017	*DCDC1, DNAJC24, IMMP1L, ELP4, PAX6, RCN1*	2.9
DMS114-PR4	chr11: 3051684-332126017	*DCDC1, DNAJC24, IMMP1L, ELP4, PAX6, RCN1*	3.1
DMS114-PR1	chr11: 35160851-36669704	*CD44, SLC1A2, PAMR1, FJX1, TRIM44, LDLRAD3, COMMD9, PRR5L, TRAF6, RAG1, RAG2, C11orf74*	2.1
DMS114-PR3	chr11: 35160851-36669704	*CD44, SLC1A2, PAMR1, FJX1, TRIM44, LDLRAD3, COMMD9, PRR5L, TRAF6, RAG1, RAG2, C11orf74*	2.4
DMS114-PR4	chr11: 35160851-36669704	*CD44, SLC1A2, PAMR1, FJX1, TRIM44, LDLRAD3, COMMD9, PRR5L, TRAF6, RAG1, RAG2, C11orf74*	2.4
DMS114-PR3	chr13:103249389-106118680	*TPP2, METTL21C, C13orf27, KDELC1, BIVM, ERCC5, SLC10A2,*	2.4
DMS114-PR3	chrX: 18274968-22292385	*From SCML2 to ZNF645*	2.3
DMS114-PR1	chrX: 96502714 -114882336	*From DIAPH2 to PLS3*	2.2
**ID**	**Peak region**	**Genes within regions with decreased copy number**	**MEAN FC**
DMS114-PR1	chr4: 75673251-79518567	*From BTC to ANXA3*	0.48
DMS114-PR1	chrY	all genes	0
DMS114-PR3	chrY	all genes	0
DMS114-PR4	chrY	all genes	0

Only regions with mean n-fold change (FC) >2 (increased copy number) or <0.5 (decreased copy number) were selected.

Supplementary Table 5: List of fusions found in the different cells

CELL ID	Gene 1	CHROMOSOME	POSITION	Gene 2	CHROMOSOME	POSITION
DMS114	*ZFAND3*	chr6	37897774	*C6orf89*	chr6	36882059
DMS114	*KLHL15*	chrX	24012796	*EIF2S3*	chrX	24078298
DMS114	*KLHL15*	chrX	24024423	*EIF2S3*	chrX	24078298
DMS114	*SUDS3*	chr12	118825300	*ENSG00000249129*	chr5	177398635
DMS114_PR1	*ZFAND3*	chr6	37897773	*C6orf89*	chr6	36882058
DMS114_PR1	*HYAL2*	chr3	50356386	*TMEM115*	chr3	50392977
DMS114_PR1	*IMMP1L*	chr11	31480752	*PAMR1*	chr11	35454198
DMS114_PR1	*DCDC5*	chr11	30942931	*CD44*	chr11	35236460
DMS114_PR1	*DENND5A*	chr11	9241081	*IPO7*	chr11	9406393
DMS114_PR1	*ENSG00000250859*	chr5	126847434	*HNRNPK*	chr9	86585718
DMS114_PR1	*ENSG00000226505*	chr2	70329652	*MRPL36*	chr5	1799905
DMS114_PR1	*TEX101*	chr19	43905187	*ARL8B*	chr3	5222492
DMS114_PR3	*ZFAND3*	chr6	37897774	*C6orf89*	chr6	36882059
DMS114_PR3	*ATP9A*	chr20	50286535	*ZFP64*	chr20	50715125
DMS114_PR3	*DCDC5*	chr11	30942931	*CD44*	chr11	35236460
DMS114_PR3	*ATP6V1C1*	chr8	104063371	*ENSG00000253320*	chr8	103989814
DMS114_PR3	*ORC3*	chr6	88321965	*ENSG00000254471*	chr11	79699535
DMS114_PR3	*ENSG00000226505*	chr2	70329652	*MRPL36*	chr5	1799905
DMS114_PR3	*TEX101*	chr19	43905187	*ARL8B*	chr3	5222492
DMS114_PR4	*ZFAND3*	chr6	37897773	*C6orf89*	chr6	36882058
DMS114_PR4	*HYAL2*	chr3	50356386	*TMEM115*	chr3	50392977
DMS114_PR4	*HMGA2*	chr12	66232348	*TBK1*	chr12	64868009
DMS114_PR4	*DCDC5*	chr11	30942931	*CD44*	chr11	35236460
DMS114_PR4	*ATP6V1C1*	chr8	104063371	*ENSG00000253320*	chr8	103989814
DMS114_PR4	*CTBP2*	chr10	126799558	*WLS*	chr1	68628714
DMS114_PR4	*ENSG00000250859*	chr5	126847434	*HNRNPK*	chr9	86585718
DMS114_PR4	*ENSG00000226505*	chr2	70329652	*MRPL36*	chr5	1799905
DMS114_PR4	*TEX101*	chr19	43905187	*ARL8B*	chr3	5222492

Supplementary Table 6: Overview of the coding and nonsynonymous SNVs, found in at least 10% of reads, in at least one of the DMS114-R cells, compared with the DMS114-P. See Supplementary_Table_6

Supplementary Table 7: List of the 5,000 genes with the most variable methylation values at CpG sites. See Supplementary_ Table_7
